# Aetiological Factors of Running-Related Injuries: A 12 Month Prospective “Running Injury Surveillance Centre” (RISC) Study

**DOI:** 10.1186/s40798-023-00589-1

**Published:** 2023-06-13

**Authors:** Aoife Burke, Sarah Dillon, Siobhán O’Connor, Enda F. Whyte, Shane Gore, Kieran A. Moran

**Affiliations:** 1 School of Health and Human Performance, Dublin City University, XG08, Lonsdale Building, Glasnevin Campus, Dublin, Ireland; 2 Insight SFI Research Centre for Data Analytics, Dublin City University, Dublin, Ireland; 3 Centre for Injury Prevention and Performance, Athletic Therapy and Training, Dublin City University, Dublin, Ireland

**Keywords:** Running, Injury, Risk factors, Kinematics, Impact acceleration, Training

## Abstract

**Background:**

Running-related injuries (RRIs) are a prevalent issue for runners, with several factors proposed to be causative. The majority of studies to date are limited by retrospective study design, small sample sizes and seem to focus on individual risk factors in isolation. This study aims to investigate the multifactorial contribution of risk factors to prospective RRIs.

**Methods:**

Recreational runners (*n* = 258) participated in the study, where injury history and training practices, impact acceleration, and running kinematics were assessed at a baseline testing session. Prospective injuries were tracked for one year. Univariate and multivariate Cox regression was performed in the analysis.

**Results:**

A total of 51% of runners sustained a prospective injury, with the calf most commonly affected. Univariate analysis found previous history of injury < 1 year ago, training for a marathon, frequent changing of shoes (every 0–3 months), and running technique (non-rearfoot strike pattern, less knee valgus, greater knee rotation) to be significantly associated with injury. The multivariate analysis revealed previous injury, training for a marathon, less knee valgus, and greater thorax drop to the contralateral side to be risk factors for injury.

**Conclusion:**

This study found several factors to be potentially causative of injury. With the omission of previous injury history, the risk factors (footwear, marathon training and running kinematics) identified in this study may be easily modifiable, and therefore could inform injury prevention strategies. This is the first study to find foot strike pattern and trunk kinematics to relate to prospective injury.

**Supplementary Information:**

The online version contains supplementary material available at 10.1186/s40798-023-00589-1.

## Key points

- One in two runners sustained a prospective running-related injury during a 12 month surveillance period, with the calf most commonly affected.
- Running technique factors such as non-rearfoot strike pattern, less knee valgus, greater knee rotation and greater thorax drop to the contralateral side relate to prospective running-related injuries.
- Training-related risk factors for injury which warrant caution include training for a marathon and frequent changing of footwear, however, these factors are easily modifiable for runners.

## Background

The proposed benefits of running are vast, with millions of runners worldwide improving their cardiovascular, musculoskeletal and psychological health with participation [1]. However, the activity of running has proven to be costly for nearly 2 out of every 3 runners, with consistently high running-related injury (RRI) prevalence rates reported [2, 3]. Overuse injuries to the knee (e.g. patellofemoral pain syndrome), shin (e.g. medial tibial stress syndrome), calf (e.g. Achilles tendinopathy) and foot (e.g. plantar fasciitis) appear to be the most common RRIs [3], typically resulting from cumulative loads that exceed the structural capacity of various tissues [4]. RRIs have been found to cause an average time-loss of 4 weeks [5], with this restriction often associated with a financial cost to the runner, in addition to a potential deterioration of cardiovascular and emotional health [5]. For this reason, several studies have sought to determine the aetiological factors of RRIs.

Several risk factors have been proposed to relate to RRIs, with sex [2, 6], age [7, 8], impact loading [9, 10], running technique [11, 12], training behaviour [8, 13] and previous history of injury [8, 14] all thought to be influential. Thus, it is critical to examine all factors and how their combined interaction may impact the occurrence of prospective RRIs.

There are perhaps five limiting factors to the previous research. Firstly, it is predominantly retrospective in nature, with few studies examining the effects of trunk (*n* = 1) [15], pelvis (*n* = 1) [15], hip (*n* = 2) [11, 16], knee (*n* = 4) [2, 11, 16, 17], and foot (*n* = 3) [2, 11, 18] kinematics prospectively. Only one prospective study has investigated the effects of impact acceleration on RRIs [19]. Secondly, some of the prospective studies are underpowered by virtue of small sample size [11, 16, 18–20], whereby the low sample size may risk the observed value not being representative of the population of interest. Thirdly, while it is well recognised that aetiological factors appear to be multifactorial in nature [4, 21], studies have focused on specific risk factors in isolation (e.g. impact loading only) [9, 22], or have concentrated on limited segments of the kinematic chain [2], which may overlook the interdependent contributions of various segments such as the pelvis or trunk to prospective injury. It is important to consider multiple aspects of internal load (e.g. impact acceleration, joint kinematics) and external load (training-related factors (e.g. distance, speed). Fourthly, the results of prospective research to date have largely involved force plate data collection which limits analysis to 3–10 strides. A recent study has identified that at least 20 consecutive strides should be utilized for stable kinematic motion capture and spatiotemporal analysis [23]. Although the precision of impact loading and kinematic motion analysis is strongest within a laboratory, the recent implementation of inertial measurement units for impact loading analysis should facilitate the examination of more representative strides, while also allowing a more insightful examination of segmental loading with simultaneous kinematic analysis. Lastly, the conflicting definitions of injury amongst the prospective RRI research has made comparisons between studies challenging, with none of the aforementioned prospective studies utilizing a consensus-based definition of RRI to date [2, 9, 22].

Thus, the aim of this study was to investigate the multifactorial contribution and interaction of impact loading, kinematic (foot, ankle, knee, hip, pelvis and trunk) and training-related factors that contribute towards prospectively injured recreational runners during a 12 month period.

## Methods

### Study Design

The Running Injury Surveillance Centre (RISC) Study was a 12 month prospective longitudinal trial of 310 recreational runners based in the greater Dublin area of Ireland (NCT03671395 www.clinicaltrials.gov). The study was approved by the Dublin City University Research Ethics Committee (DCUREC/2017/186), and informed consent was obtained from all participants prior to participation. The study was performed in accordance with the standards of ethics outlined in the Declaration of Helsinki,

### Participants

Male and female recreational runners aged over 18 years, who ran a minimum of 10 km per week for the preceding 6 months [24], were recruited from local running clubs, running events, radio advertising and social media recruitment drives between January and August 2018. Participants were excluded if they were currently injured or had sustained an injury within the 3 months prior to testing [25], had a history of cardiovascular illness, previous reconstructive joint surgery or joint replacements, or were pregnant. Study researchers (AB and SD) gave eligible participants an overview of the study, and collected baseline demographic, anthropometric, training behaviour, injury history and biomechanical data during a baseline testing session. A running-related injury definition was adapted from a consensus statement, and was defined as “any running-related (training or competition) muscle, bone, tendon or ligament pain in the lower back/legs/knee/foot/ankle that caused a restriction or stoppage of running (distance, speed, duration or training) for at least 7 days or 3 consecutive scheduled training sessions, or that required the runner to consult a physician or other health professional” [26, 27]. As lower back pain may typically be as a result of occupational or work environment stress [28], the participants were asked to only report lower back pain if it appeared to be solely as a result of running activity, and if the pain progressively worsened when completing running-based training.

Participants were asked to contact researchers if they had sustained an injury. Participants were also contacted via email or phone every fortnight for a period of 12 months from the date of their baseline session, to ensure they were still training regularly, and to determine the occurrence of any running-related injuries (RRIs) that may not have been reported immediately. If participants became injured, their injury was assessed by a Certified Athletic Therapist (AB) or a Chartered Physiotherapist (SD) to establish a diagnosis. If participants were unable to attend an injury assessment, details of the injury were taken via phone call and details of any evaluation by a healthcare professional was sought. Injured runners were tracked until their return to activity, and were subsequently tracked for further injuries until the 12 month surveillance period had ended. Participants who had an acceptable response rate (> 80%, [29]) through the 12 month surveillance period were included in the final analysis.

### Instruments

#### Survey

Participants completed an online survey prior to baseline testing. The online survey was developed based on pre-existing research that explored lifestyle and training factors relating to RRIs [13]. Face validity of the survey was conducted by a group of four experts with epidemiological and aetiological research experience, and it was then piloted with a group of 30 physically active males and females.

The final survey (Additional file 1: Material 1) comprised of 3 sections with a total of 26 questions, presented as a mix of multiple choice and open ended responses. Satellite questions were automatically prompted to gather a more detailed response to index questions where relevant. Section A of the survey consisted of 3 questions capturing the unique ID, age and sex of the participants. Section B contained 21 questions comprising of training-related questions focussing on their history of training (years running experience, participation in non-running related exercise classes), the purpose of training (motivating factors, events) and their typical training parameters (e.g. distance, speed, frequency of session, surface, footwear, presence of a niggle, experience of delayed onset of muscle soreness, execution of warm-ups, cool downs and recovery sessions). In order to document the presence of a niggle during running training, participants were asked to report and describe any “nagging pain or complaint in your lower back/lower limbs that did not restrict your training”. The final section (Section C) was made up of two main questions acquiring information on their running-related injury history (number of RRIs, location, type, duration, medical advice sought, rehabilitation completion, exacerbation or recurrence of re-injuries). Prior to any physical testing, the primary researchers checked the survey responses for accuracy and completion, with all injury and training behaviour responses clarified with participants.

#### Anthropometrics

Height (cm) (Leicester Height Measure, SECA, UK) and body mass (kg) (SECA, UK) were recorded. Leg length was then measured, which was the length (cm) between the Anterior Superior Iliac Spine and the Medial Malleolus [30]. Ankle width and knee width were measured using a callipers, and data were subsequently entered into Vicon Nexus (Vicon Motion Systems, Oxford, UK) to fulfil modelling requirements.

#### Biomechanical Analysis

Three-dimensional kinematic analysis was used to assess running technique. A 17-camera vantage motion capture system (Vicon Motion Systems, Oxford, UK) set to sample at 200 Hz. Two high speed video cameras, sampling at 100 Hz were placed 4 m behind of and perpendicular to the treadmill for visual interpretation of their running technique, if required. Thirty-two reflective markers, 14 mm in diameter, were placed on bony landmarks of the trunk, pelvis and lower limbs according to a Plug in Gait model (Vicon Motion Systems, Oxford, UK), with additional markers placed on the anterior aspects of the mid-tibia and mid-thigh bilaterally. Rigid body segments of the thorax, pelvis, thigh, shank and foot, and the joint angles between these segments were defined by the Vicon Plug in Gait modelling routine in Nexus 2 (Vicon Motion Systems, Oxford, UK). Functional joints were calculated using the ‘OSSCA’ method. Hip joint centre and the functional knee axes were calculated using the symmetrical centre of rotation estimation (SCoRE) [31] and the symmetrical axis of rotation approach (SARA) [32], respectively. Soft tissue artefact was minimized using the optimal common shape technique (OCST) [31]. Stance data at discrete time points were extracted from 90 strides for analysis (Table 1). Foot strike pattern was determined by the foot contact angle at initial contact. Foot contact angles > 8.0° were classified as rearfoot strike (RFS) pattern, < − 1.6° a forefoot strike (FFS) pattern, and − 1.6° to 8.0° represented a midfoot strike (MFS) pattern [33]. As numbers in the MFS and FFS groups were lower, these groups were combined to form a non-rearfoot strike pattern group [34].

**Table 1:** Time points for extracted stance phase variables

Stance phase variable	Definition
Initial contact	Angle when the foot makes contact with the ground
Maximum/Peak angle	Maximum angle achieved during stance
Minimum angle	Minimum angle achieved during stance
Toe off	Angle when the foot leaves the ground
Excursion	Maximum–minimum angle during stance
Angle at peak knee flexion	Angle when the knee reaches peak knee flexion

Inertial measurement units (Shimmer3 IMU, Shimmer™, Ireland) containing accelerometers were used to capture the peak (Peak_accel_) and rate (Rate_accel_) of impact acceleration of the tibia bilaterally, as well as for the sacrum, at a sampling rate of 512 Hz. These two locations were specifically chosen as they have previously been shown to be reliable methods of measuring lower limb impact loading in runners [35]. All sensors were synchronised with each other and with the motion analysis system. Two inertial measurement units were attached to the tibia bilaterally, 5 cm proximal to the medial malleolus using Hypafix® tape adhered directly to the skin, with the y-axis aligned with the long axis of the tibia [36]. The sacrum sensor was held in place within a custom-made elastic belt, with the longitudinal axis aligned to the vertical midline of the S2 spinous process [37]. This was secured further by an elastic waistband and tape. Applying tape and supportive wrapping to sensors has previously been found to capture more accurate impact acceleration data [38]. Participants wore their normal running shoes.

### Procedure

Once all reflective markers and IMUs had been attached to the body, participants completed a 5 min warm-up consisting of dynamic stretches for the hamstrings, quadriceps, hip flexors, hip extensors and calf muscle groups [39]. Running trials were conducted on a treadmill (Flow Fitness, Runner DTM3500i, The Netherlands) at a fixed speed of 2.5 m/s. The fixed speed of 2.5 m/s was chosen to allow for comparison of kinematics and impact acceleration without the confounding factor of variations in speed affecting the participants’ technique. This speed represented the average five-kilometre time of runners in the greater Dublin area, determined from the average speed reported on the Dublin Park Run database (www.parkrun.ie/events). Participants ran at 2.5 m/s for 6 min to ensure familiarisation to treadmill running [40].

### Data Processing

Motion capture data was filtered using a fourth-order zero lag 15 Hz Butterworth filter with a cut-off frequency of 15 Hz. Data were visually screened for outliers by observing entropy and amplitude using a custom-built MATLAB script (Mathworks Inc., Natick, MA, USA). Data were then processed using MATLAB to calculate the biomechanical variables of interest. Data in the sagittal plane of the foot and in the three planes of movement (sagittal, frontal, transverse) were obtained for all other segments of both limbs (ankle, knee, hip, pelvis and trunk) during the gait cycle at initial contact, time of peak knee flexion and toe-off. Maximum, minimum and excursion values per stride for each segment/joint were also recorded.

**Fig. 1:**
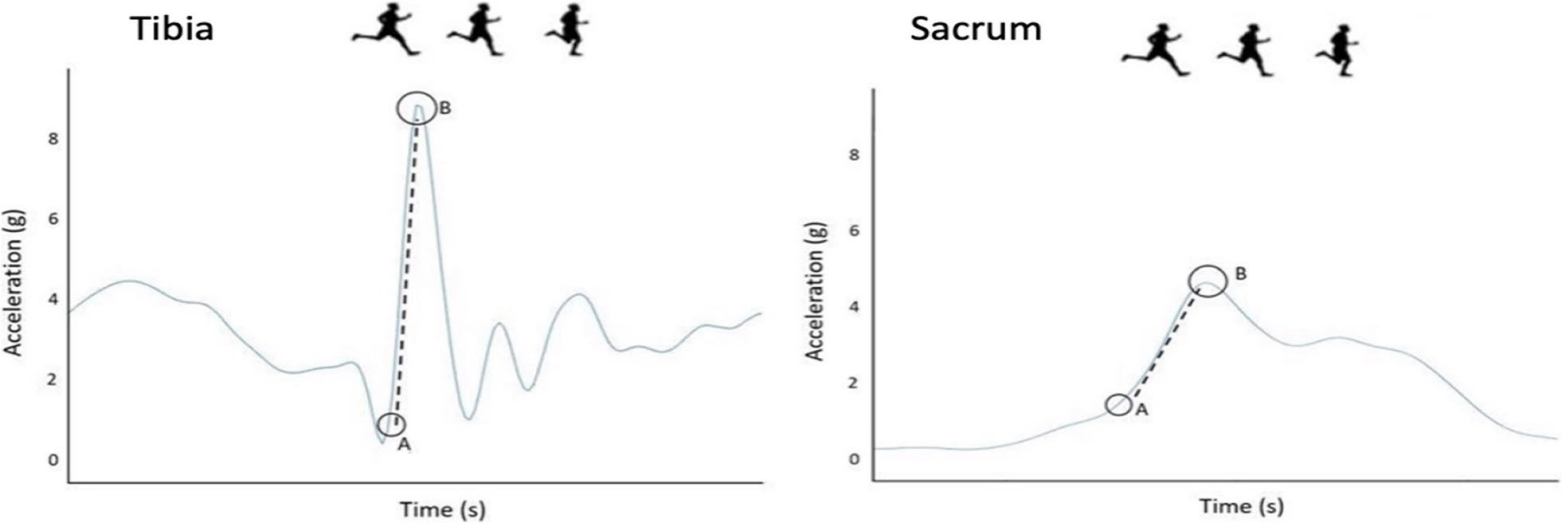
Trace of Peak_accel_ and Rate_accel_ for the shank (left) and sacrum (right). **A**: initial contact detected; dotted line - - - -: Rate_accel_, which was calculated as the slope of the peak (**B**)

Peak_accel_ and Rate_accel_ of the tibia and sacrum were processed using a custom-built MATLAB script (Mathworks Inc., Natick, MA, USA). A fourth-order, zero lag 60 Hz Butterworth filter was applied to the data and dropped packets were filled using a cubic spline. Peak_accel_ was taken as the maximal amplitude of the accelerometer’s transient at initial contact and was expressed in units of standard gravity (g = 9.8 m/s^2^). Rate_accel_ was calculated as the Peak_accel_ divided by the time to Peak_accel_ [41] (Fig. 1). Consecutive foot-strikes, taken immediately after the 6-min familiarization, were processed on both limbs.

An average of 90 strides for each limb were examined. Consistent with previous research, multiple imputation was utilized to generate multiple plausible datasets at random for dropped data packets [42]. These datasets were analysed separately and pooled at the end. In this procedure, 20 imputed datasets were generated using SPSS and pooled using Rubin’s rules [43]. In order to validate the imputation accuracy, a second imputation trial was completed where known data were deleted from two participants [42]. A subsequent independent t-test revealed no statistical difference between original data and imputed data (*p* > 0.05).

### Statistical Analysis

All statistical analyses were performed using SPSS (IBM Corp, IBM SPSS Statistics for Windows, Version 27.0, Armonk, NY). Descriptive statistics were calculated for baseline demographics, with frequencies assessed for categorical variables, and means and standard deviations for continuous variables. Boxplots were utilized to identify outliers in the kinematic and kinetic datasets. Outliers were defined as values > 1.5 times the interquartile range away from the median [44], and these were removed from the data prior to statistical analysis of differences between the groups. For runners who sustained an RRI, the limb that was injured was used in the analysis. If a runner had sustained multiple RRIs, the limb that sustained the first RRI was used. Where runners had not sustained an RRI, a random selection of their uninjured limbs was chosen. This selection was conducted at the end of the 12-month surveillance, where a percentage of injured group dominant and non-dominant limbs were matched at random to the same percentage of uninjured group dominant and non-dominant limbs. Differences in demographic characteristics between injured and uninjured runners were initially assessed with an independent t-test for continuous measures, and a chi-squared test for categorical variables.

To evaluate the contribution of possible risk factors for RRI, Cox regression was implemented with the event defined as the participant’s first RRI, or no RRI if the participant remained uninjured during the 12 month surveillance. The event time was defined as the number of days until their first RRI (injured), or until the end of the surveillance period (uninjured). Potential RRI risk factors were first entered into a univariate Cox regression to determine the independent relationship with injury. Correlations between all potential risk factors were assessed using Spearman’s rho test. If a correlation between two factors was greater than 0.8, the risk factor with the lowest p value was chosen for the multivariate analysis. Risk factors which were found to demonstrate an independent relationship with RRI in the univariate analysis (*p* ≤ 0.25) were then entered into a multivariate Cox regression prediction model, using the backward likelihood ratio approach, with *p* ≤ 0.10 applied as a cut-off level for acceptance. Hazard ratios (HR) and the corresponding 95% confidence intervals (CI) were evaluated for the risk factors associated with RRI, with statistical significance was set at *p* < 0.05.

## Results

A total of 310 recreational runners volunteered to participate in this study. Fifty-two participants were removed from the final analyses for the following reasons: sustained a non-running-related injury (e.g. work based or road traffic accident injury) (*n* = 14), had impact acceleration or kinematic data that were considered as outliers (*n* = 11), developed a long-term illness (*n* = 10), had poor response rates through the surveillance period (*n* = 10), became pregnant (*n* = 3), participated in other team-based sports (*n* = 3), or had stopped running (*n* = 1). Therefore, a total of 258 runners (163 males and 95 females) were considered for the final analyses.

### Baseline Characteristics

Demographic and anthropometric characteristics for these participants can be viewed in Table 2. There were significantly more runners with a history of previous injury in the injured group (48%) compared to the uninjured group (33%) (*p* = 0.01). No other differences existed between the groups for demographic characteristics.

**Table 2:** Demographic and anthropometric characteristics

	All (*n* = 258) Mean ± SD	Injured (*n* = 132) Mean ± SD	Uninjured (*n* = 126) Mean ± SD	*P* value
Age (years)	43.3 ± 8.9	43.5 ± 8.3	43.1 ± 9.5	0.74
Height (m)	1.7 ± 1.0	1.7 ± 0.1	1.7 ± 0.1	0.72
Weight (kg)	72.9 ± 13.1	72.2 ± 12.8	73.5 ± 13.4	0.41
BMI (kg/m^2^)	24.1 ± 3.0	24.0 ± 2.9	24.3 ± 3.1	0.39
Average training speed (km/hr)	11.4 ± 1.7	11.6 ± 1.7	11.3 ± 1.8	0.24
Annual quarterly mileage (km)	421.3 ± 283.9	420.6 ± 279.6	422.1 ± 289.3	0.97
Previous injury in past 12 months (yes)	*n* = 106 (41%)	*n* = 64 (48%)	*n* = 42 (33%)	0.01*

*n*: sample size; SD: standard deviation; m: metres; kg: kilograms; BMI: body mass index; kg/m^2^: kilogram per metres squared; km/hr: kilometres per hour; km: kilometres; *: significant *p*-value at *p* < 0.05

### RRI Prevalence

**Fig. 2:**
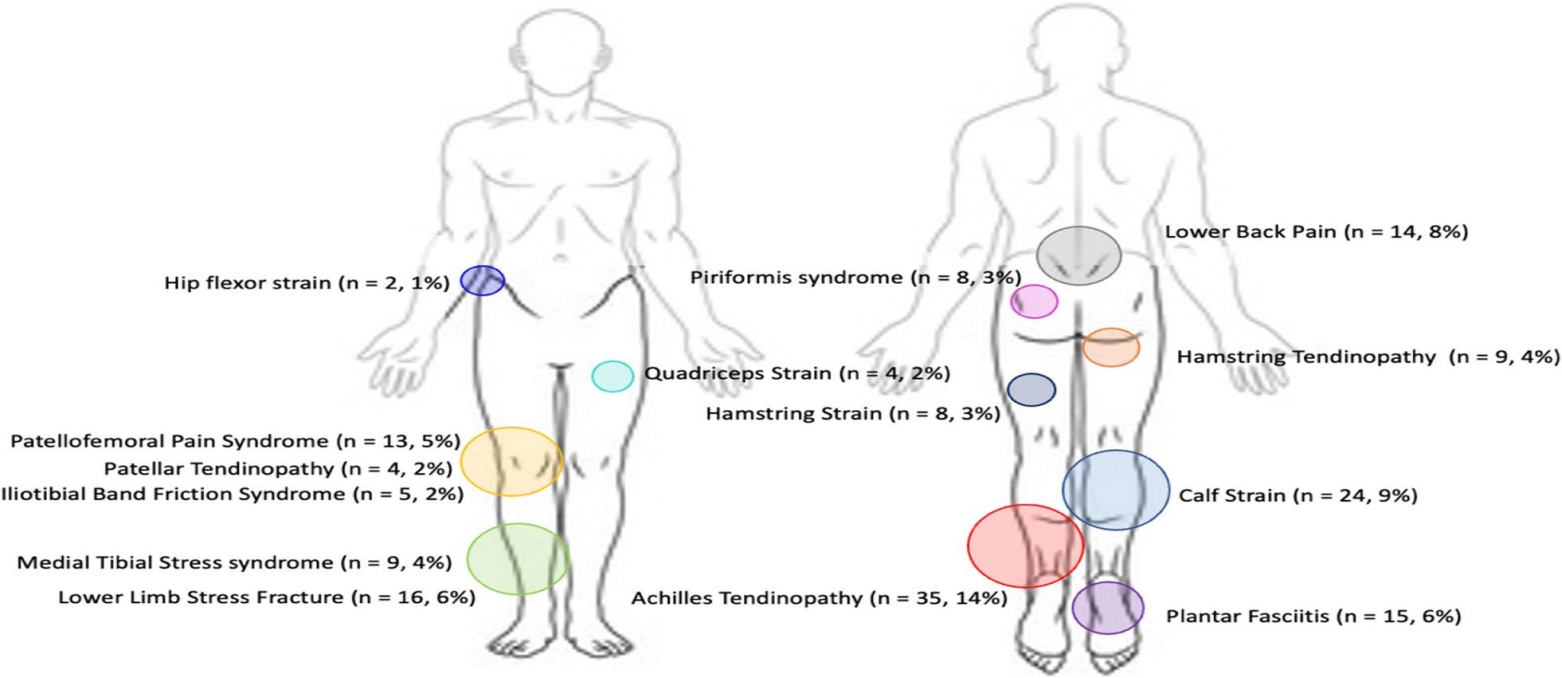
Running-related injury pathologies

One hundred and thirty-two runners (51%) sustained a total of 166 RRIs during the 12-month surveillance period. Eighty-five males (52%) and forty-seven females (50%) sustained at least one prospective RRI, with no statistical difference between sexes. A breakdown of the RRIs by pathology can be seen in Fig. 2. Achilles tendinopathy (14%), calf strains (9%) and lower back pain (8%) were the three most common pathologies experienced by all runners. The mean time-loss from injury was 50.3 ± 68.8 days (Range: 4–364 days).

Calf strain (14%) and Achilles tendinopathy (14%) were the most common injuries suffered by males, while Achilles tendinopathy (13%), lower limb stress fracture (5%) and hamstring tendinopathy (5%) were the most common injuries sustained by females (Table 3). Males were significantly more likely to have sustained a calf strain compared to females (*p* = 0.01), but no other differences were found between sexes.

**Table 3:** Running-related injury pathology by sex

	All (*n* = 258: 100%)	Males (*n* = 163: 63%)	Females (*n* = 95: 37%)	*P* value
Achilles tendinopathy	*n* = 35 (21%)	*n* = 23 (14%)	*n* = 12 (13%)	0.70
Calf strain	n = 24 (15%)	*n* = 22 (14%)	*n* = 2 (2%)	0.01*
Lower limb stress fracture	*n* = 16 (10%)	*n* = 11 (7%)	*n* = 5 (5%)	0.67
Plantar fasciitis	*n* = 15 (9%)	*n* = 11 (7%)	*n* = 4 (4%)	0.55
Lower back pain	*n* = 14 (8%)	*n* = 11 (7%)	*n* = 3 (3%)	0.48
Patellofemoral pain syndrome	*n* = 13 (8%)	*n* = 11 (7%)	*n* = 2 (2%)	0.22
Hamstring tendinopathy	*n* = 9 (5%)	*n* = 4 (3%)	*n* = 5 (5%)	0.29
Medial tibial stress syndrome	*n* = 9 (5%)	*n* = 5 (3%)	*n* = 4 (4%)	0.58
Hamstring strain	*n* = 8 (5%)	*n* = 7 (4%)	*n* = 1 (1%)	0.28
Piriformis syndrome	*n* = 8 (5%)	*n* = 5 (3%)	*n* = 3 (3%)	0.69
Iliotibial band friction syndrome	*n* = 5 (3%)	*n* = 3 (2%)	*n* = 2 (2%)	0.68
Quadriceps strain	*n* = 4 (2%)	*n* = 3 (2%)	*n* = 1 (1%)	0.62
Patellar Tendinopathy	*n* = 4 (2%)	*n* = 1 (1%)	*n* = 3 (3%)	0.17
Hip flexor strain	*n* = 2 (1%)	*n* = 1 (1%)	*n* = 1 (1%)	0.69

*n*: sample size; *: significant Chi-square *p*-value between males and females at *p* < 0.05

### Risk Factors for RRI

Means and standard deviation of demographic, impact acceleration and kinematic variables for injured and uninjured runners, in addition to differences between injury groups, can be viewed in Additional file 1: Material 2.

The univariate Cox regression analysis showed that having a previous history of injury < 1 year ago, training for a marathon, frequent changing of shoes (every 0–3 months) (Table 4), and running technique (non-rearfoot strike pattern, lower knee valgus at initial contact, lower knee valgus at toe off, lower peak knee valgus angle, greater knee internal rotation at peak knee flexion, and greater knee internal–external rotation excursion observed in injured runners compared to uninjured runners) to be significantly associated with prospective injury (*p* < 0.05) (Table 5). After adjusting for sex, age and mileage, all factors remained significant with the exception of foot strike pattern. Upon post-hoc examination, it was determined that the addition of mileage as a covariate resulted in non-rearfoot strike becoming insignificant (*p* = 0.11). In addition, greater peak thorax drop to the contralateral side became a significant univariate factor after adjusting for sex, age and mileage (*p* < 0.05). A full outline of univariate analysis findings can be seen in Additional file 1: Material 3.

**Table 4:** Univariate Cox regression findings for demographic and training-related factors

Variable	Injured (n = 132) Mean ± SD	Uninjured (n = 126) Mean ± SD	Unadjusted HR	95% CI Lower to Upper	*P* value	Adjusted HR	95% CI Lower to Upper	*P* value
Female sex (Male is reference)	47 females (50%)	48 females (50%)	0.93	0.65 to 1.33	0.71			
Age (years)	43.5 ± 8.3	43.1 ± 9.5	1.00	0.98 to 1.02	0.98			
Weight (kg)	72.2 ± 12.8	73.5 ± 13.4	0.99	0.98 to 1.01	0.38	0.99	0.97 to 1.00	0.11
Height (m)	1.7 ± 0.1	1.7 ± 0.1	0.69	0.11 to 4.19	0.68	0.25	0.18 to 3.44	0.30
BMI (kg/m^2^)	24.0 ± 2.9	24.3 ± 3.1	0.97	0.92 to 1.03	0.36	0.96	0.90 to 1.03	0.24
Annual quarterly mileage (km)	420.6 ± 279.6	422.1 ± 289.3	1.00	1.00 to 1.00	0.77			
Training Speed (km/hr)	11.6 ± 1.7	11.3 ± 1.8	1.06	0.96 to 1.16	0.27	1.06	0.96 to 1.17	0.28

kg: kilogram; m: metre; kg/m2: kilogram per metre squared; km: kilometre; km/hr: kilometres per hour; n: sample size; SD: standard deviation; HR: hazard ratio; CI: confidence interval. The adjusted results are statistically controlled for sex, age and mileage

**Table 5:** Significant univariate Cox regression findings

Variable	Injured (n = 132)	Uninjured (n = 126)	Unadjusted HR	95% CI	*P* value	Adjusted HR	95% CI	*P* value
	Mean ± SD	Mean ± SD		Lower to Upper			Lower to Upper	
FSP–RFS (Reference)	71 RFS (54%)	79 RFS (70%)	1.00			1.00		
FSP–NRFS	61 NRFS (46%)	35 NRFS (30%)	1.14	1.00 to 2.06	0.05*	1.37	0.93 to 2.01	0.11
Knee Valgus at Initial Contact (°)	− 1.6 ± 2.9	− 2.6 ± 2.8	1.09	1.03 to 1.16	0.00*	1.10	1.03 to 1.17	0.00*
Knee Valgus at Toe Off (°)	− 2.7 ± 3.0	− 3.8 ± 3.1	1.09	1.03 to 1.15	0.00*	1.10	1.04 to 1.17	0.00*
Peak Knee Valgus (°)	− 1.0 ± 3.0	− 1.9 ± 3.0	1.07	1.01 to 1.13	0.02*	1.08	1.02 to 1.15	0.01*
Knee Int Rot at Peak Knee Flexion (°)	21.3 ± 7.5	19.5 ± 8.0	1.03	1.00 to 1.05	0.03*	1.03	1.00 to 1.05	0.04*
Knee Rotation Excursion (°)	20.3 ± 5.2	19.3 ± 4.1	1.04	1.00 to 1.08	0.03*	1.05	1.01 to 1.09	0.02*
Peak Thorax Drop to Contralateral Side (°)	1.2 ± 2.3	0.8 ± 2.2	1.06	0.98 to 1.15	0.13	1.09	1.00 to 1.18	0.05*
No previous injury (Reference)	68 (52%)	84 (67%)	1.00			1.00		
Previous Injury	64 (48%)	42 (33%)	1.57	1.12 to 2.21	0.01*	1.57	1.10 to 2.23	0.01*
Not training for a marathon (Reference)	48 (46%)	70 (54%)	1.00			1.00		
Training for a marathon	84 (64%)	56 (46%)	1.75	1.22 to 2.50	0.00*	1.76	1.22 to 2.54	0.00*
Change shoes 0–3 months (Reference)	14 (11%)	13 (10%)	1.00			1.00		
Change shoes 4–6 months	40 (30%)	37 (29%)	0.50	0.23 to 1.07	0.07	0.49	0.23 to 1.06	0.07
Change shoes 7–12 months	42 (32%)	33 (26%)	0.46	0.22 to 0.98	0.05*	0.45	0.20 to 0.99	0.05*
Change shoes 12 months +	36 (27%)	43 (34%)	0.40	0.19 to 0.86	0.02*	0.38	0.17 to 0.85	0.02*

FSP: foot strike pattern; RFS: rear-foot strike; NRFS: non-rear-foot strike; Int Rot: internal rotation; *n*: sample size; SD: standard deviation; HR: hazard ratio; CI: confidence interval; *: *p* value significant at *p* < 0.05. The adjusted results are statistically controlled for sex, age and mileage

With respect to the multivariate Cox regression analysis, only four variables remained in the final model (Table 6), with two of these being statistically significant (*p* < 0.05). A lower knee valgus at toe off in injured runners compared to uninjured runners (HR: 1.09; 95% CI 1.03 to 1.16, *p* = 0.01) and training for a marathon (HR: 1.47; 95% CI 1.01 to 2.24, *p* = 0.04) were both found to be significant risk factors for prospective injury. Thorax drop to contralateral side and previous history of injury < 1 year ago were also significant contributors to the final multivariate model.

**Table 6:** Results of the multivariate Cox regression

Variable	Injured (*n* = 132)	Uninjured (*n* = 126)	HR	95% CI	*P* value
	Mean ± SD	Mean ± SD		Lower to Upper	
Knee Valgus at Toe Off (°)	− 2.7 ± 3.0	− 3.8 ± 3.1	1.09	1.03 to 1.16	0.006*
Thorax Drop to Contralateral Side (°)	1.2 ± 2.3	0.8 ± 2.2	1.08	1.00 to 1.17	0.063
No previous injury (Reference)	68 (52%)	84 (67%)	1.00		
Previous Injury	64 (48%)	42 (33%)	1.57	1.41 to 2.04	0.069
Not training for a marathon (Reference)	48 (46%)	70 (54%)	1.00		
Training for a marathon	84 (64%)	56 (46%)	1.47	1.01 to 2.14	0.043*

*n*: sample size; SD: standard deviation; HR: hazard ratio; CI: confidence interval; *: *p* value significant at *p* < 0.05

## Discussion

This discussion primarily compares and contrasts the findings of this study with prospective research, where possible. The prioritising of prospective comparisons over retrospective comparisons is because of the unclear cause and effect differentiation that retrospective research presents. It is feasible that where a smaller value for a variable is evident in the injured group of a retrospective study, it is a compensatory response for a larger value in the injured group causing the injury (as would be evident in a prospective study), and vice versa.

### Injury Prevalence

The one year injury prevalence of 51% is similar to previous studies [19, 45]. The calf was the most commonly injured region, supporting a trend which has been observed previously [19, 46, 47]. The knee has often been found to be the most commonly injured region within running epidemiology research [2, 48, 49], but was the second most popular location in this study. Authors are uncertain why this may be, but propose that the greater prevalence of non-rearfoot strike runners (46%) observed in the injured group of this study may indicate greater posterior lower leg complex loading [50], compared to the patellofemoral joint load that is observed in rearfoot strike runners [50, 51]. Limited studies in the past have reported the pathology of injury, making comparisons limited. The most common injuries in this study were Achilles tendinopathy, calf strain, lower limb stress fracture and plantar fasciitis, findings which support that of previous research [11, 52–54].

### Potential Risk Factors for RRI

#### Demographic Characteristics

Intrinsic risk factors such as sex, age and anthropometry have been well researched in RRIs. Although the present study found males to suffer significantly more calf injuries than females, there was no significant effect for sex on overall injury in the Cox regression model. This is in support of Satterwaite et al. [7], who also noted males to be at greater risk of calf injuries. The evidence for sex as a risk factor for RRI is conflicting however, with some studies suggesting males to be at greater risk of injury [7, 55], some proposing that females are at greater risk [2], and some finding no risk associated with either sex [56–59]. It has been speculated that injury risk may differ between sexes due to the differences in anatomical (femoral inclination and femoral anteversion) [60–62], physiological (heart and lung size and capacity) [63] and biomechanical (joint kinematics and landing strategies) [64–66] characteristics of males and females, however the basis for such differences is largely theoretical to date.

Regarding increasing age, some studies have found deficits to flexibility, strength, bone density, and proprioception [54]. These physiological changes along with a reduced capacity for healing and recovery could suggest an increase in susceptibility to prospective injuries for an older athlete [54, 67]. The present study however did not find age to relate to injury, which adds further support to previous findings [2, 56, 59]. With respect to anthropometrics, body mass index (BMI) is one of the most popular measures utilised within research, as it is considerate of both height and weight. It has been proposed that a greater BMI would result in excessive loading or forces on the lower extremities [68]. The present study supports the findings of several others having found no association between BMI and RRIs [2, 48, 69].

#### Previous History of Injury and Training-Related Factors

The present study found that having an injury within the previous year increased the odds of sustaining a prospective injury by 1.57 times, a finding that adds further validation to systematic reviews in the area [8, 70]. When returning from previous injury, there may be incomplete healing of the original injury [8], which may cause permanent and long-lasting structural or biomechanical mal-adaptations, increasing the chances of subsequent re-injuries [71]. To compound this, if rehabilitation was insufficient in terms of addressing predisposing intrinsic (strength, mobility, flexibility, impact loading) and extrinsic (load, speed, footwear) risk factors for the injury, the return to full participation may be at a compromised level resulting in potentially dysfunctional movement and coordination strategies [72, 73]. This may overload previously vulnerable or weak structures and again, tissue failure may result [70].

With regards to training-related factors, the present study found that training for a marathon was significantly associated with a 1.76 greater risk of injury, reinforcing the findings of Macera et al. [74]. Marathon runners generally prepare for the event with periodical increments in training mileage. To date there are inconsistent findings regarding mileage, with some authors noting significantly lower training volumes in marathon runners [75, 76], and other studies reporting significantly higher training volumes in marathon runners [14, 77]. The present study found no effect of mileage on RRIs, a finding that supports the majority of research in this area [2, 48, 69, 78, 79]. A potential reason for the lack of clarity may be that most studies capture absolute mileage at a single point in time, and subsequently relate this to injury. While this method is logistically and financially advantageous for researchers, it does not consider the change in mileage over time and therefore may not identify sharp increases or changes in training volume. Recent systematic reviews have advocated for the implementation of the exponentially weighted moving average model, a variant of the acute: chronic workload ratio, which considers training volume on an ongoing basis, and is more likely to inform of deleterious training loads that may cause injury in non-contact sports [80, 81].

With regards to footwear, the present study found that infrequent changing of running shoes is protective of injury, suggesting that those who change shoes less frequently (> 3 months) to be at lesser risk of injury. This finding lends further support to Taunton et al*.* [82], who too reported a significantly lower risk for injury in males who had infrequent shoe changes (4–6 months) compared to a change every 1–3 months. A frequent change of shoes may increase the risk of injury particularly if the shoes are of a different brand, model or cushioning. These changes may alter the foot position (e.g. foot strike pattern) thereby changing the distribution of loading within the lower extremity [83], and subsequently injuring unfamiliar with the associated overload [4, 84].

Regarding training speed, the present study did not find speed to relate to injury, a finding that is akin to previous prospective research [2, 79, 85]. Although greater speeds increase the loading on the body [86–88], it is possible that the increase in general running speed is slow enough over time (due to the slow rate of physiological anaerobic adaptations) that the body has time to adapt to the associated increase in loading.

#### Impact Acceleration

The present study did not find any association between injury and either the Peak_accel_ or Rate_accel_. Only one study to date has investigated the association between impact acceleration and prospective injury, and similar to our findings, observed no significant differences in sacrum peak acceleration between injured and uninjured runners [19]. Although retrospective research has found a potential relationship between higher tibial acceleration and tibial stress fractures in female runners [44, 89, 90], it is unclear whether the high loading was a cause or an effect of the lower limb stress fractures in these studies. In addition, these retrospective studies may have found a link due to the investigation of specific RRI injuries local to the segment that they examined [44, 89, 90], as opposed to general overuse RRIs collectively. Although this injury specific approach may be insightful for runners with a history of a specific injury, it does not inform injury prevention practices for the majority of runners who generally will not know what specific injury they need to protect against, particularly if they have not sustained an injury previously.

#### Running Kinematics

Regarding running kinematics, there were significant associations found between injury and both knee and thorax kinematics. Less knee valgus was associated with injury in the present study, with lower valgus ankles observed in injured runners compared to uninjured runners. This is important as only one prospective study appears to have previously examined this, and although less peak knee valgus angles were observed in injured runners, their finding was not significant [11]. This lack of significance however, may have been due to an underpowering of their statistical analysis associated with the low number of injured participants (*n* = 12). Evidently, there is a lack of research in the area of knee kinematics and prospective injury in runners. Authors have postulated that extreme or excessive valgus and varus knee positions increase the load bearing on the medial and lateral knee [91, 92], which may lead to high patellofemoral stress, overloading of the articular cartilage and subchondral bone [93], in addition to increased strain on the iliotibial band [94]. However, these extreme and excessive knee positions have only been observed in retrospective studies to date, which may therefore indicate a compensatory action as a result of a previous knee injury.

The present study also found greater knee internal rotation at peak knee flexion and greater knee rotation excursion in injured runners compared to uninjured runners. This provides new evidence for knee kinematics and RRIs, with no prospective studies previously investigating knee internal rotation at peak knee flexion. Furthermore, only one prospective study has assessed knee rotation excursion, reporting no difference between injured and uninjured runners [17]. This may have been due to an underpowered sample size of injured runners (*n* = 10), or due to methodological differences, whereby Hein et al*.* [17] examined Achilles tendon injuries only. It has been hypothesized that greater knee internal rotation and greater knee rotation excursion may cause an increase in pressure and load at the patellofemoral joint [95, 96], and that a lack of control of these motions is thought to play an important role in the development of patellofemoral pain syndrome [97].

Regarding trunk kinematics, greater peak thorax drop to the contralateral side was demonstrated in injured runners compared to uninjured runners. Again, the present study adds new evidence to this area with only one study previously examining thorax kinematics and prospective RRIs [15]. Shen et al. [15] found no differences in peak trunk flexion and peak trunk ipsilateral flexion between injured and uninjured runners, although their sample size was likely underpowered (*n* = 15). The thorax and upper body account for approximately 60% of a person’s total body mass [98], and therefore trunk motion likely influences loading [99]. Thorax drop to the contralateral side has been found to be a normal aspect of gait in healthy subjects [100], and the motion is due to the activity of the oblique abdominal muscles [101]. This intricate interplay of thorax and pelvic kinematics and musculature allows runners to minimize centre of mass displacement [100]. However, the inability to control excessive thorax drop and other trunk motion has been suggested to lead to excessive stress on the pelvis [102] and lower limb such as the calf muscle complex [103] or knee [104], and as a result may overload susceptible tissues leading to injury. It also appears that a lack of core strength and endurance may result in an inability to control trunk motion during running [105], which has subsequent effects at the hip and knee [106]. This may explain why studies have focussed on core strength interventions in the rehabilitation of running injuries [107, 108].

An additional finding of interest (although not significant in the adjusted analysis) was that non-rearfoot strike runners were more likely to have sustained a prospective injury than rearfoot strike runners, a finding that is similar to the results of Hollander et al*.* [109] and Dingenen et al. [110]. A non-rearfoot strike pattern is thought to invoke greater loading on the plantarflexor muscles and Achilles tendon [50, 111, 112], which aligns well with the calf and Achilles tendon being the most commonly injured sites in the present study. This is the first prospective study to examine foot strike technique in its categorical form, with previous studies assessing continuous measures of foot contact angle [11] and strike index [2, 18] only. As eluded to in a recent systematic review, perhaps the investigation of foot strike technique via continuous measures is not sensitive enough to differentiate the loading differences that exist between rearfoot and non-rearfoot strike runners [113], and that examining discrete foot strike patterns (non-rearfoot versus rearfoot strikes) is more relevant.

#### Multivariate Analysis

Four variables contributed to the final model, with two of these being significant. Multivariate analyses typically suggest factors that interact with each other to explain injury [114]. Thus, it may be important to consider these variables in combination rather than in isolation. Less knee valgus at toe off and training for a marathon were both found to be significant risk factors for prospective injury. In practice, it may be pertinent to consider load management when training for a marathon. While knee kinematics may require effort to adjust, training load is a more modifiable mediator in this instance and as a result, may be a useful consideration for injury prevention strategies. Although thorax drop to the contralateral side and previous history of injury < 1 year ago were not significant in the final multivariate model, they too are important factors to consider within the greater picture, given their presence in the final model. Having a previous history of injury is not modifiable, but it can help to identify runners who may be more susceptible upon returning to participation. Therefore, runners who have a history of injury within the past year should take measures to ensure effective rehabilitation and injury preventative training.

### Clinical Implications

A number of factors were identified that increased the risk of prospective injury in this study. Consistent with previous research, having a history of injury appears to be one of the greatest risk factors for future injury. Clinically, healthcare professionals and biomechanists should strive to prescribe appropriate and effective rehabilitation, to ensure the runners can regain tissue strength and capacity to tolerate training loads again. The present study also found training for a marathon to be a risk for injury, and perhaps runners should be made aware of this when considering their commitment to the event. It has been advised that runners should build a solid foundation of running fitness, followed by gradual increases in running volume incorporating various speeds and distances [115].

Running kinematics were also found to relate to injury, factors which may be effectively altered with running retraining programmes [116]. Several studies have reported significant reductions in pain [117–119] and injury occurrence [120] with running retraining, with some demonstrating long-term efficacy in maintaining kinematic [121] and impact acceleration changes [122] over 8 to 12 months respectively.

### Study Limitations

This study has five main limitations. Firstly, data pertaining to impact acceleration, kinematics and training were obtained at one point in time prior to injury occurrence, and it is therefore unknown how consistent these factors would have been throughout the 1 year surveillance period, and if these factors might have changed with fatigue or over-training. For greater accuracy and application, future studies should perhaps consider more frequent assessment, or even run-by-run assessment. Secondly, the kinetic and kinematic data was collected during treadmill running, which may not be reflective of the training surface that the participants typically train on [123, 124]. Thirdly, running technique was assessed for a relatively short period of time, which may not have been considerate of the typical duration that runners usually train for, and subsequently the influence of fatigue on biomechanics and potential injury is limited. Fourthly, runners ran at a fixed speed of 9 km/hr, which may have been slower or faster than their typical training pace, and as a result may have influenced their natural gait. However, running speed has been shown to affect both impact acceleration and kinematics [125], and the aim for a fixed speed in the present study was to control for this effect amongst a large cohort of runners [9, 22]. Lastly, injuries in the present study were investigated collectively as general overuse RRIs. This was conducted with a view to inform injury prevention strategies going forward, as determining the risk factors for RRIs collectively will attend to the greater running community more effectively than establishing the risk factors for specific RRIs individually.

## Conclusion

This prospective study provided further clarity to the body of evidence suggesting that RRIs are multifactorial in nature. Training-related risk factors that proved significant included training for a marathon and frequent changing of footwear (every 0–3 months), factors that are easily managed from an injury avoidance perspective. In terms of running technique, this is the first study to find evidence for a relationship between non-rearfoot strike pattern and prospective injury risk, highlighting the importance of categorical foot strike analysis. Other kinematics which indicated heightened injury risk included lesser knee valgus, greater knee rotation and greater thorax drop to the contralateral side, all significant factors which have not been well investigated with respect to prospective injury previously. Lastly, the present study further supported the significance that having a history of injury increased future injury risk; clearly indicating the need for careful return to participation practices.

Further large scale prospective research should seek to consider more frequent or on-going (e.g. run-by-run) analyses of impact acceleration, kinematics and training load through the prospective trial period.

## Supplementary Information

**Additional file 1**.

## Data Availability

The datasets generated and/or analysed during the current study are not publicly available due to EU GDPR Regulations, but are available from the corresponding author on reasonable request.

## Abbreviations

BMI: Body mass index
CI: Confidence interval
cm: Centimetre
FFS: Forefoot strike
FSP: Foot strike pattern
g: Gravity
HR: Hazard ratio
Hz: Hertz
IMU: Inertial measurement unit
kg: Kilogram
kg/m^2^: Kilogram per metre squared
km: Kilometre
km/hr: Kilometres per hour
m: Metre
MFS: Midfoot strike
mm: Millimetre
m/s: Metres per second
n: Sample size
OCST: Optimal common shape technique
OSSCA: Combination of OCST, SCoRE and SARA approach
NRFS: Non-rearfoot strike
Peak_accel_: Peak acceleration
Rate_accel_: Rate of acceleration
RISC: Running injury surveillance centre
RFS: Rearfoot strike
RRI: Running-related injury
SARA: Symmetrical axis of rotation
SCoRE: Symmetrical centre of rotation estimation
SD: Standard deviation
