# Supplementary material for: Aetiological Factors of Running-Related Injuries: A 12 Month Prospective “Running Injury Surveillance Centre” (RISC) Study
Source: Sports Med Open. 2023 Jun 13;9:46. doi: 10.1186/s40798-023-00589-1 (PMC10264338; doi:10.1186/s40798-023-00589-1)
Supplement: Supplementary file 1 — Additional file 1. [file 40798_2023_589_MOESM1_ESM.docx]

Supplementary information for Aetiological factors of running-related injuries: A 12 month prospective “Running Injury Surveillance Centre” (RISC) Study

Additional file 1: Material 1. RISC Study Survey

**RISC Study Survey**

***Section A - Demographics***

Q.1. What is your unique ID number?

*Open-ended response.*

Q.2. What age are you?

*Open-ended response [Numerical].*

Q.3. Please select your gender.

- *Male*
- *Female*
- *Prefer not to say*

***Section B - Training***

Q.4. Do you attend any exercise classes? Please tick all that apply.

- *Yoga*
- *Pilates*
- *Aerobics*
- *Dance/Zumba*
- *Spinning*
- *Altitude Chamber*
- *Boxercise*
- *HIIT (High Intensity Interval Training)*
- *S&C (Strength & Conditioning)*
- *TRX*
- *CrossFit*
- *Swimming*
- *MMA*
- *Other (Please specify)*
- *No I don’t attend exercise classes*

Q.4. (a) How many times per week do you attend exercise classes?

- *1 time per week*
- *2 times per week*
- *3 times per week*
- *4 times per week*
- *5 times per week*
- *6 times per week*
- *7 times per week*
- *7+ times per week*

Q.5. Do you regularly go to the gym? Please tick no if you go to the gym for the purpose of group exercise classes.

- *Yes, 1-2 times per week*
- *Yes, 3-4 times per week*
- *Yes, 5-7 times per week*
- *No, I don’t go to the gym*

Q.5. (a) What does a typical gym session consist of for you? Please tick all that apply.

- *Cardiovascular (e.g. Rowing, Cross Trainer, Swimming, Bike)*
- *Strength (e.g. Free Weights, Weight Machines)*
- *Flexibility (e.g. Stretching)*
- *Plyometrics (e.g. Hops, Jumps, Box Jumps)*
- *Other (Please specify)*

Q.6. Since you first started running training, what is the total amount of years that you have trained? (Please do not include years when you did not train regularly e.g. taking a year out).

- *6-12 months*
- *1-2 years*
- *3-5 years*
- *6-10 years*
- *11-15 years*
- *15+ years*

Q.7. Do you run throughout the year or on a seasonal basis?

- *Throughout the year*
- *Seasonal basis*

Q.7. (a) If you ticked “seasonal basis”, how many months of the year do you run?

- *1 month*
- *2 months*
- *3 months*
- *4 months*
- *5 months*
- *6 months*
- *7 months*
- *8 months*
- *9 months*
- *10 months*
- *11 months*

Q.8. What is the purpose of running for you? Please tick all that apply.

- *Fitness*
- *Physique*
- *Enjoyment*
- *Mental health*
- *Train for competition*
- *To accomplish a personal goal*
- *Social interaction*
- *Convenience*
- *Other (Please specify)*

Q.8. (a) Please rank in order of importance the purpose of running for you. e.g. 1= primary purpose.

*Select number from drop-down menu beside each respective motivation.*

Q.9. Do you have any running related events that you are currently training for or that you plan to train for within the next year? Please tick all that apply.

- *5km*
- *10km*
- *Mini-marathon*
- *10 mile*
- *Half-marathon*
- *¾ marathon*
- *Marathon*
- *Ultra-marathon*
- *Ironman*
- *Duathlon*
- *Triathlon*
- *Cross-country*
- *Adventure race*
- *Trail/Mountain race*
- *Organised track and field event*
- *Other (Please specify)*
- *I am not training for a running related event*

Q.10. On average, how many times per week do you run?

- *1 time per week*
- *2 times per week*
- *3 times per week*
- *4 times per week*
- *5 times per week*
- *6 times per week*
- *7 times per week*
- *7+ times per week*

Q.11. At present, what distance (kilometres) per week do you run?

*Open-ended response [Numerical].*

Q.12. How many kilometres collectively have you ran over the  course of the last three months?

*Open-ended response [Numerical].*

Q.13. What is your average running pace? (km/hr) If you are unsure, please refer to pace graph provided.

*Open-ended response [Numerical].*

Q.14. Do you regularly increase the intensity of running training week to week?

- *Yes*
- *No*
- *I am unsure*

Q.14. (a) How do you increase the intensity of running training from week to week? Please tick all that apply.

- *Increase distance*
- *Increase pace*
- *Increase the number of running sessions*
- *Change gradient*
- *Do tempo runs*
- *Other (Please specify)*

Q.15. Do you include any of the following sessions as part of your running training? Please tick all that apply.

- *Interval training*
- *Speed work*
- *Hill running*
- *Fartlek*
- *Other (Please specify)*

Q.16. What surface do you run on most often? If you run on multiple surfaces for an equal number of sessions, please tick those that apply.

- *Road*
- *Grass*
- *Footpath*
- *Track*
- *Sand*
- *Treadmill*
- *Astroturf*
- *Other (Please specify)*

Q.17. How often do you change running shoes?

- *Every 0-3 months*
- *Every 4-6 months*
- *Every 7-12 months*
- *Every 12+ months*

Q.18. Do you wear insoles or insole devices in your running shoes? (Arch support, heel lift, etc.)

- *Yes, they were prescribed to me*
- *Yes, I bought them in a shop*
- *Yes, my shoes are manufactured with a specific arch support/shock absorption feature*
- *No, I don’t wear insoles or insole devices*
- *I am unsure*

Q.19. Do you apply any strapping/taping/braces/supports before going for a run? (Please do not include orthotic devices/insoles).

- *Yes, always*
- *Yes, sometimes*
- *No, I don’t apply strapping/taping/braces/supports*
- *I am unsure*

Q.19. (a) What location of the body do you apply strapping/taping/support/brace to? Please tick all that apply.

- *Lower back*
- *Sacroiliac joint*
- *Hip*
- *Inner thigh*
- *Buttock*
- *Front of thigh*
- *Back of thigh*
- *Outer thigh*
- *Knee*
- *Shin*
- *Calf*
- *Ankle*
- *Foot*
- *Heel*
- *Toes*

Q.20. Do you currently have any persistent or nagging pain or complaint in your lower back/lower limbs that you experience while running but does not restrict your training?

- *Yes*
- *No*
- *I am unsure*

Q.20. (a) Please give details of this persistent pain (e.g. you may describe the location, type, severity, duration, etc.).

*Open-ended response [Text].*

Q.21. Delayed Onset of Muscle Soreness (DOMS) is a muscular pain/ache following a session of increased intensity or unfamiliar activity. The soreness typically lasts 24-72 hours. Do you experience DOMS?

- *Yes, typically once a week*
- *Yes, typically once a fortnight*
- *Yes, typically once a month*
- *Yes, typically multiple times per year*
- *No, I do not experience DOMS*
- *I am unsure*

Q.22. Do you usually warm up before a running session?

- *Yes, always*
- *Yes, sometimes*
- *No, I do not usually warm-up*
- *I am unsure*

Q.22. (a) What does your warm up consist of? Please tick all that apply.

- *Static stretch*
- *Dynamic stretch*
- *Cardiovascular*
- *Foam rolling*
- *Plyometrics*
- *Joint mobility*
- *Other (Please specify)*

Q.23. Do you usually warm down/cool down after a running session?

- *Yes, always*
- *Yes, sometimes*
- *No, I do not usually warm-up*
- *I am unsure*

Q.23. (a) What does your warm down/cool down normally consist of? Please tick all that apply.

- *Static stretch*
- *Dynamic stretch*
- *Cardiovascular*
- *Foam rolling*
- *Massage*
- *Swimming*
- *Other (Please specify)*

Q.24. Do you include any recovery sessions as part of your training? A recovery session is a planned session where the objective is to re-establish an optimal state for training (e.g. rest, massage, light cardio, baths).

- *Yes, always*
- *Yes, sometimes*
- *No, I do not usually warm-up*
- *I am unsure*

Q.24. (a) Which of the following are included in your recovery session? Please tick all that apply.

- *Rest*
- *Foam rolling*
- *Stretching*
- *Light run*
- *Cycle*
- *Swim*
- *Cryotherapy*
- *Hot baths*
- *Massage*
- *Light resistance training*
- *Other (Please specify)*

***Section C – Running-related Injury***

Q.25. Have you ever experienced a running- related injury? ( A running related injury is any muscle, bone tendon or ligament pain that caused you to stop running/restricted your running (either your speed, distance or duration) and lasted 7 days or three consecutive training sessions/ required you to seek a physician or health care practitioner.)

- *Yes*
- *No*
- *I am unsure*

Q.26. Have you had any previous running related injuries in the past 2 years? A running-related injury is any muscle, bone, tendon or ligament pain in the lower back/legs/knee/foot/ankle that caused you to stop running/ restricted your running (either your distance, speed, duration or training)

**AND**

1. lasted at least 7 days or 3 consecutive scheduled training sessions

**OR**

1. required you to consult a physician or other health care professional.

- *Yes, I had a lower back/lower limb running-related injury that lasted at least 7 days or 3 scheduled training sessions (i).*
- *Yes, I had a lower back/lower limb running-related injury that required me to consult a physician or other healthcare professional (ii).*
- *Yes, I had a lower back/lower limb running-related injury that lasted at least 7 days or 3 scheduled training sessions (i)* ***AND*** *that required me to consult a physician or other healthcare professional (ii).*
- *No, I have not has any lower back/lower limb running-related injury in the past 2 years.*
- *I am unsure.*

Q.26. (a) How many lower back/lower limb running-related injuries have you had in the past 2 years?

- *1 running-related injury*
- *2 running-related injuries*
- *3 running-related injuries*
- *4 running-related injuries*
- *5 running-related injuries*
- *5+ running-related injuries*

Q.26. (a)(i) Thinking of one of these back/lower limb running-related injuries in the past 2 years please select the location of the body that you had this injury.

- *Lower back*
- *Sacroiliac joint*
- *Hip*
- *Inner thigh*
- *Buttock*
- *Front of thigh*
- *Back of thigh*
- *Outer thigh*
- *Knee*
- *Shin*
- *Calf*
- *Ankle*
- *Foot*
- *Heel*
- *Toes*

Q.26. (a)(ii) What month did this injury occur?

*Select month from drop-down menu.*

Q.26. (a)(iii) What year did this injury occur?

*Select year from drop-down menu.*

Q.26. (a)(iv) Still thinking of this injury, what type of injury was it?

- *Cut/Graze*
- *Contusion/Bruise*
- *Ligament tear/Sprain (e.g. twisted ankle)*
- *Subluxation/Dislocation*
- *Broken bone/Fracture (*****NOT*** *a stress fracture)*
- *Cartilage/Meniscus/Labrum injury*
- *Stress fracture*
- *Muscle strain/tear/rupture*
- *Tendon injury*
- *Nerve injury*
- *Shin splints type pain (*****NOT*** *a stress fracture)*
- *Bursitis*
- *Fat pad aggravation*
- *Blisters*
- *Other (Please specify)*

Q.26. (a)(v) Still thinking of this injury, did you miss any training because of it?

- *No, I did not miss training*
- *Yes, I missed less than 7 days*
- *Yes, I missed between 7 and 28 days*
- *Yes, I missed between 1 and 6 months*
- *Yes, I missed more than 6 months*
- *I am unsure*
- *Other (Please specify)*

Q.26. (a)(vi) Still thinking of this injury, did you require any medical advice? Please tick all that apply.

- *No, I did not require any medical advice*
- *Yes, I got medical advice from an internet resource*
- *Yes, I received medical advice from my coach*
- *Yes, I received medical advice from my GP/doctor*
- *Yes, I received medical advice from a medical professional (Chartered Physiotherapist, Certified Athletic Therapist, Physical Therapist, Chiropractor, Osteopath)*
- *I had to go to A&E*
- *I received medical advice from a family member or fried who is not a medical professional*

Q.26. (a)(vii) Still thinking of this injury, did you complete a rehabilitation programme after the injury? A rehabilitation programme usually involves completing a set of exercises that have been specifically tailored to your injury.

- Yes, I was given once by a medical professional *(Doctor, Chartered Physiotherapist, Certified Athletic Therapist, Physical Therapist, Chiropractor, Osteopath)*
- *Yes, I rehabilitated the injury myself*
- *No, I did not need a rehabilitation programme*
- *I am unsure*
- *Other (Please specify)*

Q.26. (a)(viii) Still thinking of this injury, do you feel you have recovered fully from this injury?

- *Yes*
- *No*
- *I am unsure*

Q.26. (a)(ix) Still thinking of this injury, has there been any exacerbation or re-injury of this in the past 2 years? Exacerbation refers to the worsening of your initial injury before it was fully recovered. Re-injury refers to a recurring injury after your initial injury had recovered.

- *Yes, I had a re-injury at the same location and of the same type*
- *Yes, I have had an exacerbation at the same location and of the same type*
- *Yes, I had a re-injury at the same location and of a different type*
- *Yes, I have had an exacerbation at the same location and of a different type*
- *I am unsure*
- *No, I have not had any exacerbations or re-injuries*

Q.26. (a)(ix)(1) How soon after the initial injury did the exacerbation occur?

- *Within 2 months*
- *Between 2 and 12 months*
- *Between 12 and 24 months*
- *I am unsure*

Q.26. (a)(ix)(2) How soon after the initial injury did the re-injury occur?

- *Within 2 months*
- *Between 2 and 12 months*
- *Between 12 and 24 months*
- *I am unsure*

Additional file 1: Material 2. Kinetic and Kinematic Variable Means and Standard Deviation, with Independent T-Test and Univariate Cox Regression Findings.

|  | | | Injured | | Uninjured | Injured v Uninjured | Unadjusted HR | 95% CI | P value | Adjusted HR | 95% CI | P value |
| --- | --- | --- | --- | --- | --- | --- | --- | --- | --- | --- | --- | --- |
|  | | | **Mean ± SD** | | **Mean ± SD** | **P value** |  | **Lower to Upper** |  |  | **Lower to Upper** |  |
| Demographics | | |  | |  |  |  |  |  |  |  |  |
| Age (years) | | | 43.5 ± 8.3 | | 43.1 ± 9.5 | 0.74 | 1.00 | 0.98 to 1.02 | 0.98 |  |  |  |
| Weight (m) | | | 72.2 ± 12.8 | | 73.5 ± 13.4 | 0.41 | 0.99 | 0.98 to 1.01 | 0.38 | 0.99 | 0.97 to 1.00 | 0.11 |
| Height (kg) | | | 1.7 ± 0.1 | | 1.7 ± 0.1 | 0.72 | 0.69 | 0.11 to 4.19 | 0.68 | 0.25 | 0.18 to 3.44 | 0.30 |
| BMI (kg/m^2^) | | | 24.0 ± 2.9 | | 24.3 ± 3.1 | 0.39 | 0.97 | 0.92 to 1.03 | 0.36 | 0.96 | 0.90 to 1.03 | 0.24 |
| Average training speed (km/hr) | | | 11.6 ± 1.6 | | 11.3 ± 1.8 | 0.24 | 1.06 | 0.96 to 1.16 | 0.27 | 1.06 | 0.96 to 1.17 | 0.28 |
| Annual quarterly mileage (km) | | | 420.6 ± 279.6 | | 422.1 ± 289.3 | 0.97 | 1.00 | 1.00 to 1.00 | 0.77 |  |  |  |
| Impact Acceleration | | |  | |  |  |  |  |  |  |  |  |
| Tibia Peak_accel_ (g) | | | 5.9 ± 2.5 | | 5.7 ± 2.3 | 0.46 | 1.03 | 0.96 to 1.10 | 0.43 | 1.03 | 0.96 to 1.11 | 0.44 |
| Tibia Rate_accel_ (g/s) | | | 317.4 ± 233.7 | | 301.4 ± 231.3 | 0.59 | 1.00 | 1.00 to 1.00 | 0.56 | 1.00 | 1.00 to 1.00 | 0.55 |
| Sacrum Peak_accel_ (g) | | | 5.1 ± 2.6 | | 4.8 ± 2.4 | 0.41 | 1.04 | 0.97 to 1.11 | 0.30 | 1.03 | 0.96 to 1.11 | 0.44 |
| Sacrum Rate_accel_ (g/s) | | | 494.6 ± 321.2 | | 487.7 ± 288.7 | 0.86 | 1.00 | 1.00 to 1.00 | 0.84 | 1.00 | 1.00 to 1.00 | 0.79 |
| *Kinematics* | | |  | |  |  |  |  |  |  |  |  |
| *Initial Contact (°)* |  |  | |  | | | | | | | | |
| Foot Dorsiflexion | | | 10.4 ± 6.2 | | 11.2 ± 6.4 | 0.28 | 0.99 | 0.96 to 1.01 | 0.31 | 0.99 | 0.96 to 1.02 | 0.43 |
| Ankle Eversion | | | 1.8 ± 2.3 | | 1.7 ± 1.9 | 0.65 | 1.04 | 0.95 to 1.13 | 0.42 | 1.04 | 0.94 to 1.14 | 0.47 |
| Ankle Dorsiflexion | | | 9.0 ± 5.1 | | 8.9 ± 5.2 | 0.85 | 1.00 | 0.97 to 1.04 | 0.89 | 1.01 | 0.97 to 1.05 | 0.72 |
| Ankle External Rotation | | | -7.0 ± 9.2 | | -6.5 ± 7.6 | 0.65 | 0.99 | 0.97 to 1.01 | 0.46 | 0.99 | 0.97 to 1.02 | 0.58 |
| Knee Valgus | | | -1.6 ± 2.9 | | -2.6 ± 2.8 | 0.01* | 1.09 | 1.03 to 1.16 | 0.00* | 1.10 | 1.03 to 1.17 | 0.00* |
| Knee Flexion | | | 18.0 ± 4.4 | | 17.0 ± 4.3 | 0.11 | 1.03 | 0.99 to 1.07 | 0.19 | 1.02 | 0.98 to 1.07 | 0.34 |
| Knee Internal Rotation | | | 5.1 ± 6.5 | | 4.2 ± 7.1 | 0.30 | 1.02 | 0.99 to 1.04 | 0.23 | 1.02 | 0.99 to 1.04 | 0.29 |
| Hip Adduction | | | 8.8 ± 3.4 | | 9.7 ± 4.0 | 0.06 | 0.96 | 0.91 to 1.00 | 0.06 | 0.95 | 0.90 to 1.00 | 0.03* |
| Hip Flexion | | | 34.6 ± 6.2 | | 34.7 ± 6.1 | 0.82 | 1.00 | 0.97 to 1.03 | 0.79 | 0.99 | 0.97 to 1.02 | 0.71 |
| Hip Rotation (+ Internal Rotation; - External Rotation) | | | -0.9 ± 6.5 | | -1.2 ± 7.0 | 0.68 | 1.01 | 0.98 to 1.03 | 0.64 | 1.01 | 0.98 to 1.04 | 0.53 |
| Pelvic Drop to Contralateral Side | | | 1.7 ± 2.2 | | 2.1 ± 2.7 | 0.19 | 0.96 | 0.89 to 1.03 | 0.25 | 0.95 | 0.89 to 1.03 | 0.19 |
| Anterior Pelvic Tilt | | | 14.3 ± 5.0 | | 14.5 ± 5.6 | 0.69 | 0.99 | 0.96 to 1.03 | 0.72 | 0.99 | 0.96 to 1.03 | 0.74 |
| Pelvis Rotation to Ipsilateral Side | | | -3.4 ± 4.0 | | -3.6 ± 3.5 | 0.71 | 1.02 | 0.97 to 1.07 | 0.57 | 1.01 | 0.96 to 1.07 | 0.70 |
| Thorax Drop to Ipsilateral Side | | | -2.6 ± 2.4 | | -2.7 ± 2.4 | 0.80 | 1.02 | 0.95 to 1.10 | 0.52 | 1.04 | 0.96 to 1.12 | 0.37 |
| Thorax Anterior Tilt | | | 8.0 ± 4.6 | | 7.3 ± 4.6 | 0.20 | 1.02 | 0.98 to 1.06 | 0.25 | 1.03 | 0.99 to 1.07 | 0.18 |
| Thorax Rotation to Ipsilateral Side | | | -12.2 ± 4.3 | | -12.4 ± 5.0 | 0.86 | 1.00 | 0.96 to 1.04 | 0.97 | 1.00 | 0.96 to 1.05 | 0.90 |
| *Peak Knee Flexion (°)* |  |  | |  | | | | | | | | |
| Foot Plantarflexion | | | -2.5 ± 1.1 | | -2.6 ± 1.7 | 0.57 | 1.04 | 0.92 to 1.17 | 0.57 | 1.04 | 0.92 to 1.18 | 0.57 |
| Ankle Eversion | | | 5.6 ± 2.5 | | 5.6 ± 2.4 | 0.96 | 1.02 | 0.95 to 1.10 | 0.63 | 1.02 | 0.94 to 1.10 | 0.66 |
| Ankle Dorsiflexion | | | 24.3 ± 3.4 | | 23.7 ± 3.6 | 0.17 | 1.01 | 0.99 to 1.05 | 0.34 | 1.02 | 0.99 to 1.05 | 0.25 |
| Ankle External Rotation | | | -21.6 ± 8.3 | | -21.1 ± 7.6 | 0.65 | 0.99 | 0.97 to 1.01 | 0.43 | 0.99 | 0.97 to 1.02 | 0.57 |
| Knee Valgus | | | -4.0 ± 3.6 | | -4.6 v 3.4 | 0.22 | 1.03 | 0.98 to 1.09 | 0.21 | 1.04 | 0.98 to 1.10 | 0.18 |
| Knee Flexion | | | 42.6 ± 4.9 | | 42.2 ± 3.9 | 0.42 | 1.02 | 0.98 to 1.07 | 0.29 | 1.02 | 0.98 to 1.07 | 0.34 |
| Knee Internal Rotation | | | 21.3 ± 7.5 | | 19.5 ± 8.0 | 0.07 | 1.03 | 1.00 to 1.05 | 0.03* | 1.03 | 1.00 to 1.05 | 0.04* |
| Hip Adduction | | | 11.9 ± 4.2 | | 12.6 ± 4.6 | 0.23 | 0.98 | 0.94 to 1.02 | 0.24 | 0.97 | 0.93 to 1.02 | 0.22 |
| Hip Flexion | | | 27.9 ± 6.5 | | 28.0 ± 7.0 | 0.95 | 1.00 | 0.98 to 1.03 | 0.98 | 1.00 | 0.98 to 1.03 | 0.91 |
| Hip External Rotation | | | -5.4 ± 6.3 | | -5.2 ± 7.1 | 0.83 | 1.00 | 0.97 to 1.02 | 0.82 | 1.00 | 0.97 to 1.03 | 0.86 |
| Pelvic Drop to Contralateral Side | | | 3.5 ± 2.7 | | 3.7 ± 2.8 | 0.61 | 0.98 | 0.92 to 1.05 | 0.61 | 0.99 | 0.92 to 1.05 | 0.66 |
| Anterior Pelvic Tilt | | | 11.8 ± 5.0 | | 11.8 ± 6.1 | 0.95 | 1.00 | 0.97 to 1.03 | 0.97 | 1.00 | 0.97 to 1.03 | 0.92 |
| Pelvis Rotation to Ipsilateral Side | | | -4.6 ± 4.0 | | -4.5 ± 3.6 | 0.88 | 0.99 | 0.95 to 1.04 | 0.81 | 0.99 | 0.94 to 1.04 | 0.57 |
| Thorax Drop to Ipsilateral Side | | | -3.9 ± 2.0 | | -4.0 ± 3.6 | 0.62 | 1.03 | 0.95 to 1.12 | 0.49 | 1.04 | 0.96 to 1.13 | 0.33 |
| Thorax Anterior Tilt | | | 9.9 ± 4.8 | | 9.3 ± 4.6 | 0.31 | 1.02 | 0.98 to 1.06 | 0.33 | 1.02 | 0.98 to 1.07 | 0.24 |
| Thorax Rotation to Ipsilateral Side | | | -4.8 ± 3.9 | | -5.1 ± 4.4 | 0.58 | 1.00 | 0.96 to 1.05 | 0.84 | 1.01 | 0.96 to 1.05 | 0.79 |
| *Toe Off (°)* |  |  | |  | | | | | | | | |
| Foot Plantarflexion | | | -49.5 ± 7.0 | | -51.3 ± 7.2 | 0.06 | 1.02 | 1.00 to 1.05 | 0.06 | 1.03 | 1.00 to 1.05 | 0.07 |
| Ankle Abduction (+ Eversion; - Inversion) | | | 0.3 ± 2.1 | | 0.2 ± 2.0 | 0.87 | 1.01 | 0.93 to 1.10 | 0.77 | 1.01 | 0.92 to 1.11 | 0.81 |
| Ankle Plantarflexion | | | -12.3 ± 5.9 | | -13.2 ± 6.1 | 0.28 | 1.01 | 0.99 to 1.05 | 0.34 | 1.02 | 0.99 to 1.05 | 0.25 |
| Ankle Rotation (+ Internal Rotation; - External Rotation) | | | -1.0 ± 8.4 | | -0.9 ± 8.2 | 0.91 | 1.00 | 0.98 to 1.02 | 0.81 | 1.00 | 0.98 to 1.02 | 0.88 |
| Knee Valgus | | | -2.7 ± 3.0 | | -3.8 ± 3.1 | 0.01* | 1.09 | 1.03 to 1.15 | 0.00* | 1.10 | 1.04 to 1.17 | 0.00* |
| Knee Flexion | | | 17.0 ± 6.6 | | 17.9 ± 6.7 | 0.32 | 0.98 | 0.96 to 1.01 | 0.19 | 0.98 | 0.95 to 1.01 | 0.16 |
| Knee Internal Rotation | | | 4.6 ± 6.1 | | 4.4 ± 7.2 | 0.86 | 1.00 | 0.98 to 1.03 | 0.75 | 1.00 | 0.98 to 1.03 | 0.75 |
| Hip Adduction | | | 1.0 ± 3.3 | | 1.5 ± 3.3 | 0.21 | 0.96 | 0.91 to 1.01 | 0.15 | 0.95 | 0.90 to 1.01 | 0.10 |
| Hip Extension | | | -3.0 ± 5.6 | | -3.2 ± 6.3 | 0.82 | 1.00 | 0.97 to 1.03 | 0.96 | 1.00 | 0.97 to 1.03 | 0.96 |
| Hip External Rotation | | | -8.1 ± 6.5 | | -8.0 ± 7.1 | 0.91 | 1.00 | 0.97 to 1.02 | 0.90 | 1.00 | 0.97 to 1.03 | 0.99 |
| Pelvic Drop to Ipsilateral Side | | | -3.8 ± 2.5 | | -3.7 ± 2.4 | 0.73 | 0.98 | 0.91 to 1.05 | 0.59 | 0.99 | 0.91 to 1.07 | 0.76 |
| Anterior Pelvic Tilt | | | 16.8 ± 4.7 | | 16.6 ± 5.7 | 0.80 | 1.01 | 0.97 to 1.04 | 0.73 | 1.01 | 0.97 to 1.04 | 0.73 |
| Pelvis Rotation to Contralateral Side | | | 2.6 ± 4.1 | | 2.4 ± 3.5 | 0.76 | 1.02 | 0.97 to 1.07 | 0.53 | 1.01 | 0.96 to 1.05 | 0.74 |
| Thorax Drop to Contralateral Side | | | 1.0 ± 2.5 | | 0.7 ± 2.2 | 0.30 | 1.05 | 0.97 to 1.14 | 0.20 | 1.07 | 0.99 to 1.16 | 0.10 |
| Thorax Anterior Tilt | | | 7.9 ± 4.9 | | 7.6 ± 4.6 | 0.69 | 1.01 | 0.97 to 1.04 | 0.76 | 1.01 | 0.97 to 1.05 | 0.64 |
| Thorax Rotation to Contralateral Side | | | 13.1 ± 4.6 | | 13.2 ± 4.3 | 0.95 | 1.00 | 0.96 to 1.04 | 0.95 | 1.00 | 0.96 to 1.05 | 0.98 |
| *Excursion/ ROM (°)* |  |  | |  | | | | | | | | |
| Foot Flexion | | | 59.9 ± 9.9 | | 62.5 ± 10.4 | 0.05* | 0.98 | 0.97 to 1.00 | 0.06 | 0.99 | 0.97 to 1.00 | 0.08 |
| Ankle Eversion | | | 6.0 ± 1.7 | | 6.2 ± 1.8 | 0.43 | 0.99 | 0.89 to 1.09 | 0.77 | 0.99 | 0.89 to 1.10 | 0.87 |
| Ankle Flexion | | | 38.9 ± 5.9 | | 39.3 ± 5.7 | 0.54 | 1.00 | 0.97 to 1.03 | 0.86 | 1.00 | 0.96 to 1.03 | 0.80 |
| Ankle Rotation | | | 22.9 ± 5.3 | | 23.0 ± 4.8 | 0.85 | 1.00 | 0.97 to 1.04 | 0.85 | 1.00 | 0.97 to 1.04 | 0.89 |
| Knee Abduction | | | 3.6 ± 1.5 | | 3.5 ± 1.4 | 0.58 | 1.04 | 0.92 to 1.17 | 0.53 | 1.06 | 0.94 to 1.21 | 0.35 |
| Knee Flexion | | | 28.8 ± 5.6 | | 28.5 ± 5.3 | 0.66 | 1.02 | 0.98 to 1.05 | 0.36 | 1.02 | 0.98 to 1.05 | 0.30 |
| Knee Rotation | | | 20.3 ± 5.2 | | 19.3 ± 4.1 | 0.11 | 1.04 | 1.00 to 1.08 | 0.03* | 1.05 | 1.01 to 1.09 | 0.02* |
| Hip Adduction | | | 11.9 ± 3.8 | | 12.2 ± 3.9 | 0.56 | 0.99 | 0.94 to 1.04 | 0.67 | 0.99 | 0.94 to 1.04 | 0.74 |
| Hip Flexion | | | 38.0 ± 5.2 | | 38.5 ± 4.8 | 0.42 | 0.99 | 0.96 to 1.02 | 0.54 | 0.99 | 0.95 to 1.03 | 0.49 |
| Hip Rotation | | | 10.5 ± 3.8 | | 10.1 ± 3.3 | 0.31 | 1.03 | 0.98 to 1.08 | 0.26 | 1.04 | 0.98 to 1.09 | 0.20 |
| Pelvic Abduction | | | 8.2 ± 2.9 | | 8.6 ± 3.1 | 0.30 | 0.97 | 0.92 to 1.04 | 0.40 | 0.96 | 0.90 to 1.03 | 0.28 |
| Pelvis Tilt | | | 7.3 ± 1.9 | | 7.4 ± 2.0 | 0.91 | 1.01 | 0.92 to 1.11 | 0.81 | 1.01 | 0.92 to 1.11 | 0.89 |
| Pelvis Rotation | | | 8.5 ± 3.5 | | 8.1 ± 3.5 | 0.48 | 1.02 | 0.97 to 1.08 | 0.36 | 1.03 | 0.97 to 1.10 | 0.29 |
| Thorax Abduction | | | 5.4 ± 2.3 | | 5.2 ± 1.6 | 0.38 | 1.05 | 0.96 to 1.15 | 0.34 | 1.06 | 0.96 to 1.17 | 0.23 |
| Thorax Tilt | | | 3.5 ± 1.3 | | 3.5 ± 1.3 | 0.92 | 1.02 | 0.89 to 1.17 | 0.78 | 1.03 | 0.89 to 1.20 | 0.71 |
| Thorax Rotation | | | 25.5 ± 6.8 | | 25.6 ± 6.4 | 0.85 | 1.00 | 0.97 to 1.03 | 0.92 | 1.00 | 0.96 to 1.03 | 0.89 |
| *Maximum/Peak Angle (°)* |  |  | |  | | | | | | | | |
| Foot Dorsiflexion | | | 10.4 ± 6.2 | | 11.3 ± 6.3 | 0.27 | 0.99 | 0.96 to 1.01 | 0.31 | 0.99 | 0.96 to 1.02 | 0.42 |
| Ankle Eversion | | | 6.1 ± 2.6 | | 6.2 ± 2.4 | 0.78 | 1.01 | 0.94 to 1.08 | 0.87 | 1.01 | 0.93 to 1.89 | 0.84 |
| Ankle Dorsiflexion | | | 26.6 ± 3.8 | | 26.2 ± 3.6 | 0.39 | 1.04 | 0.99 to 1.09 | 0.17 | 1.04 | 0.99 to 1.09 | 0.14 |
| Ankle Rotation (+ Internal Rotation; - External Rotation) | | | -0.1 ± 8.4 | | 0.3 ± 7.9 | 0.78 | 1.00 | 0.97 to 1.02 | 0.69 | 1.00 | 0.97 to 1.02 | 0.77 |
| Knee Valgus | | | -1.0 ± 3.0 | | -1.9 ± 3.0 | 0.02* | 1.07 | 1.01 to 1.13 | 0.02* | 1.08 | 1.02 to 1.15 | 0.01* |
| Knee Flexion | | | 42.7 ± 4.9 | | 42.2 ± 3.9 | 0.42 | 1.02 | 0.98 to 1.07 | 0.29 | 1.02 | 0.98 to 1.07 | 0.34 |
| Knee Internal Rotation | | | 23.0 ± 7.6 | | 21.5 v 7.7 | 0.14 | 1.02 | 1.00 to 1.05 | 0.06 | 1.03 | 1.00 to 1.05 | 0.05* |
| Hip Adduction | | | 12.7 ± 3.9 | | 13.5 ± 4.4 | 0.14 | 0.97 | 0.93 to 1.01 | 0.14 | 0.97 | 0.92 to 1.01 | 0.12 |
| Hip Flexion | | | 34.7 ± 6.2 | | 35.0 ± 6.0 | 0.77 | 1.00 | 0.97 to 1.03 | 0.78 | 0.99 | 0.97 to 1.02 | 0.71 |
| Hip Internal Rotation | | | 1.1 ± 6.2 | | 0.9 ± 6.8 | 0.80 | 1.00 | 0.98 to 1.03 | 0.76 | 1.01 | 0.98 to 1.04 | 0.65 |
| Pelvic Drop to Contralateral Side | | | 4.1 ± 2.6 | | 4.6 ± 2.7 | 0.20 | 0.96 | 0.90 to 1.03 | 0.23 | 0.96 | 0.89 to 1.03 | 0.22 |
| Anterior Pelvic Tilt | | | 17.1 ± 4.7 | | 17.0 ± 5.6 | 0.96 | 1.00 | 0.97 to 1.04 | 0.87 | 1.00 | 0.97 to 1.04 | 0.85 |
| Pelvis Rotation to Contralateral Side | | | 2.9 ± 3.9 | | 2.6 ± 3.4 | 0.55 | 1.02 | 0.97 to 1.08 | 0.37 | 1.02 | 0.96 to 1.07 | 0.55 |
| Thorax Drop to Contralateral Side | | | 1.2 ± 2.3 | | 0.8 ± 2.2 | 0.21 | 1.06 | 0.98 to 1.15 | 0.13 | 1.09 | 1.00 to 1.18 | 0.05 |
| Thorax Anterior Tilt | | | 10.6 ± 4.8 | | 10.1 ± 4.6 | 0.43 | 1.02 | 0.98 to 1.05 | 0.44 | 1.02 | 0.98 to 1.06 | 0.34 |
| Thorax Rotation to Contralateral Side | | | 13.2 ± 4.6 | | 13.2 ± 4.2 | 0.93 | 1.00 | 0.96 to 1.04 | 0.92 | 1.00 | 0.95 to 1.05 | 0.98 |
| *Minimum (°)* |  |  | |  | | | | | | | | |
| Foot Plantarflexion | | | -49.5 ± 7.0 | | -51.3 ± 7.2 | 0.06 | 1.02 | 1.00 to 1.05 | 0.07 | 1.03 | 1.00 to 1.05 | 0.07 |
| Ankle Abduction (+ Eversion; - Inversion) | | | 0.1 ± 2.1 | | -0.1 ± 2.0 | 0.73 | 1.02 | 0.94 to 1.12 | 0.64 | 1.02 | 0.93 to 1.12 | 0.70 |
| Ankle Plantarflexion | | | -12.3 ± 5.9 | | -13.2 ± 6.1 | 0.28 | 1.01 | 0.99 to 1.05 | 0.35 | 1.02 | 0.99 to 1.05 | 0.25 |
| Ankle External Rotation | | | -23.0 ± 8.2 | | -22.8 ± 7.3 | 0.86 | 0.99 | 0.97 to 1.02 | 0.58 | 1.00 | 0.97 to 1.02 | 0.69 |
| Knee Valgus | | | -4.6 ± 3.5 | | -5.4 ± 3.2 | 0.07 | 1.05 | 1.00 to 1.11 | 0.06 | 1.06 | 1.00 to 1.12 | 0.05 |
| Knee Flexion | | | 13.9 ± 4.9 | | 13.7 ±4.6 | 0.81 | 1.00 | 0.96 to 1.04 | 0.92 | 1.00 | 0.96 to 1.03 | 0.78 |
| Knee Internal Rotation | | | 2.7 ± 5.8 | | 2.2 ± 6.9 | 0.56 | 1.01 | 0.93 to 1.04 | 0.46 | 1.01 | 0.98 to 1.04 | 0.50 |
| Hip Adduction | | | 0.8 ± 3.2 | | 1.3 ± 3.2 | 0.22 | 0.96 | 0.91 to 1.02 | 0.16 | 0.95 | 0.90 to 1.01 | 0.11 |
| Hip Extension | | | -3.2 ± 5.6 | | -3.5 ± 6.1 | 0.70 | 1.00 | 0.97 to 1.03 | 0.82 | 1.00 | 0.97 to 1.04 | 0.88 |
| Hip External Rotation | | | -9.4 ± 6.0 | | -9.2 ± 6.9 | 0.76 | 1.00 | 0.97 to 1.02 | 0.77 | 1.00 | 0.97 to 1.03 | 0.83 |
| Pelvic Drop to Ipsilateral Side | | | -4.0 ± 2.4 | | -4.0 ± 2.2 | 0.92 | 0.99 | 0.92 to 1.07 | 0.78 | 1.00 | 0.91 to 1.09 | 0.93 |
| Anterior Pelvic Tilt | | | 9.7 ± 4.8 | | 9.7 ± 5.8 | 0.93 | 1.00 | 0.97 to 1.03 | 0.94 | 1.00 | 0.97 to 1.04 | 0.89 |
| Pelvis Rotation to Ipsilateral Side | | | -5.5 ± 3.9 | | -5.5 ± 3.5 | 0.94 | 1.00 | 0.95 to 1.05 | 0.99 | 0.99 | 0.94 to 1.05 | 0.77 |
| Thorax Drop to Ipsilateral Side | | | -4.2 ± 2.1 | | -4.3 ± 2.2 | 0.62 | 1.03 | 0.95 to 1.12 | 0.48 | 1.04 | 0.96 to 1.13 | 0.35 |
| Thorax Anterior Tilt | | | 7.1 ± 4.7 | | 6.6 ± 4.5 | 0.40 | 1.01 | 0.98 to 1.05 | 0.47 | 1.02 | 0.98 to 1.06 | 0.38 |
| Thorax Rotation to Ipsilateral Side | | | -12.3 ± 4.3 | | -12.4 ± 5.0 | 0.85 | 1.00 | 0.96 to 1.04 | 0.96 | 1.00 | 0.96 to 1.05 | 0.88 |

g: g force; g/s: g force per second; °: degrees; ROM: range of motion; CI: confidence interval; *: significant p value at < 0.05.

Additional file 1: Material 3. Univariate Cox regression findings for categorical variables

| **Variable** | **Unadjusted HR** | **95% CI** | **P value** | **Adjusted HR** | **95% CI** | **P value** |
| --- | --- | --- | --- | --- | --- | --- |
|  |  | **Lower to Upper** |  |  | **Lower to Upper** |  |
| RFS (Reference) | 1.00 |  |  |  |  |  |
| NRFS | 1.14 | 1.00 to 2.06 | 0.05* | 1.37 | 0.93 to 2.01 | 0.11 |
| No previous injury (References) | 1.00 |  |  |  |  |  |
| Previous Injury | 1.57 | 1.12 to 2.21 | 0.01* | 1.57 | 1.10 to 2.23 | 0.01* |
| Not training for 5km (Reference) | 1.00 |  |  |  |  |  |
| 5km | 0.77 | 0.53 to 1.11 | 0.16 | 0.77 | 0.53 to 1.12 | 0.17 |
| Not training for 10km (Reference) | 1.00 |  |  |  |  |  |
| 10km | 0.92 | 065 to 1.30 | 0.63 | 0.91 | 0.64 to 1.30 | 0.60 |
| Not training for half-marathon (Reference) | 1.00 |  |  |  |  |  |
| Half Marathon | 0.91 | 0.64 to 1.29 | 0.58 | 0.91 | 0.64 to 1.28 | 0.58 |
| Not training for marathon (Reference) | 1.00 |  |  |  |  |  |
| Marathon | 1.75 | 1.22 to 2.50 | 0.00* | 1.76 | 1.22 to 2.54 | 0.00* |
| Doesn’t do speed work (Reference) | 1.00 |  |  |  |  |  |
| Speed Work | 1.20 | 0.84 to 1.71 | 0.32 | 1.23 | 0.85 to 1.76 | 0.27 |
| Doesn’t do hill runs (Reference) | 1.00 |  |  |  |  |  |
| Hill Runs | 1.20 | 0.82 to 1.75 | 0.35 | 1.20 | 0.82 to 1.77 | 0.36 |
| Change shoes every 0-3 months (Reference) | 1.00 |  |  |  |  |  |
| Change shoes 4-6 months | 0.50 | 0.23 to 1.07 | 0.07 | 0.49 | 0.23 to 1.06 | 0.07 |
| Change shoes 7-12 months | 0.46 | 0.22 to 0.98 | 0.05* | 0.45 | 0.20 to 0.99 | 0.05* |
| Change shoes 12 months + | 0.40 | 0.19 to 0.86 | 0.02* | 0.38 | 0.17 to 0.85 | 0.02* |
| Doesn’t wear insoles (Reference) | 1.00 |  |  |  |  |  |
| Wear Insoles | 0.96 | 0.63 to 1.46 | 0.83 | 0.97 | 0.64 to 1.48 | 0.89 |
| Doesn’t experience a niggle (Reference) | 1.00 |  |  |  |  |  |
| Niggle | 1.16 | 0.81 to 1.66 | 0.42 | 1.16 | 0.81 to 1.67 | 0.42 |
| Niggle Unsure | 0.75 | 0.36 to 1.57 | 0.45 | 0.76 | 0.37 to 1.60 | 0.47 |
| Never does a warm up | 1.00 |  |  |  |  |  |
| Warm up always | 1.30 | 0.80 to 2.11 | 0.30 | 1.33 | 0.81 to 2.16 | 0.26 |
| Warm up sometimes | 1.31 | 0.80 to 2.12 | 0.28 | 1.31 | 0.81 to 2.14 | 0.27 |

RFS: rearfoot strike pattern; NRFS: non-rearfoot strike pattern; km: kilometre; HR: hazard ratio; CI: confidence interval; *: significant at p value < 0.05.
